# Estrogen replacement therapy: effects of starting age on final height of girls with chronic kidney disease and short stature

**DOI:** 10.1186/s12887-022-03406-y

**Published:** 2022-06-21

**Authors:** Davoud Amirkashani, Farzaneh Rohani, Mahmoud Khodadost, Rozita Hoseini, Hamidreza Alidoost, Sedigheh Madani

**Affiliations:** 1 Department of Pediatric Endocrinology, Aliasghar Children’s Hospital, Aliasghar Clinical Research Development Center, Iran University of Medical Sciences, Tehran, Iran; 2 Department of Epidemiology, School of Public Health, Iran University of Medical Sciences, Tehran, Iran; 3 Department of Epidemiology, School of Public Health, Larestan University of Medical Sciences, Larestan, Iran; 4 Department of Pediatrics, Faculty of Medicine, Baqiyatallah University of Medical Sciences, Tehran, Iran

**Keywords:** Growth retardation, Delayed puberty, GH treatment, Estrogen replacement therapy, Chronic kidney disease

## Abstract

**Introduction:**

We investigated the age of starting Estrogen replacement therapy as a key parameter for reaching near normal Final Height (FH) in Chronic Kidney Disease (CKD) girls with growth retardation.

**Method:**

This open label, quasi-experimental designed and matched controlled clinical trial was performed on CKD girls with short stature and later onset of puberty or delayed puberty according to clinical and laboratory investigations. Participants of group 1 and 2 had been treated with Growth Hormone (GH), and Ethinyl Estradiol (EE). EE was administered from 11 and 13 yrs. old in groups 1 and 2 respectively. Group 3 was selected from patients that did not accept to start GH or EE till 15 years old. The effect of the age of starting EE on FH, GH therapy outcomes, bone density, and calcium profile were evaluated.

**Result:**

Overall, 16, 22, and 21 patients were analyzed in groups 1, 2, and 3 respectively. Mean Mid-Parental Height (MPH) had no significant difference between the 3 groups. GH therapy significantly enhanced mean FH in groups 1 and 2 in comparison with group 3 (β = − 4.29, *p* < 0.001). Also, multivariable backward linear regression illustrated significant negative association between FH and age of starting EE (β = 0.26, *p* < 0.001). Mean Para Thyroid Hormone (PTH), mean femoral and lumbar bone density were significantly enhanced after GH and EE therapy (*p* value: < 0.001).

**Conclusion:**

We recommend starting EE from 11 yrs. old in CKD short stature girls who have no clinical and laboratory sign of sexual maturity at 11 yrs. to enhance the cost effectiveness of GH therapy.

**Supplementary Information:**

The online version contains supplementary material available at 10.1186/s12887-022-03406-y.

## What is already known about this topic?

Prevalence of short stature among patients with chronic kidney diseases is almost 50% and its currently recommended treatment, growth hormone, is very expensive. Almost always, there has been a strict view about use of estrogen in these patients because of concern about the closure of growth palate.

## What this study adds?

We investigated and propose a solution for the increment of growth hormone efficacy. We hereby show that the age of starting estrogen replacement therapy (ERT) is very important for achieving a proper spurt and better final height in these patients. Also, this investigation illustrated that postponing ERT leads to undesirable final height.

## Introduction

Short stature is an important problem in pediatric patients with chronic kidney disease (CKD) and approximately 40-50% of children with ESRD have reduced final height (FH) (<3th percentile) which increases the risk of mortality and is a marker of poor control in CKD [1, 2].

Uremic growth failure is a consequence of multiple factors such as chronic malnutrition, metabolic acidosis, multiple hormonal disturbances, water and electrolytes imbalance, and renal anemia [3]. Chronic metabolic acidosis causes Growth Hormone (GH) insensitivity and it has been known as the main hormonal disturbance that leads to growth failure of CKD children [4]. So, evidence-based guideline for GH replacement therapy has proposed to start appropriate dose of GH as soon as documenting persistent growth failure in children with stage 3-5 End Stage Renal Disease (ESRD) or on dialysis. Persistent growth failure is defined as a height below the third percentile for age and sex besides a height velocity below the 25 percentile [5].

Although GH therapy significantly increases height Standard Deviation (SD) score and also final height in children with CKD [5, 6], these children’s final height remains under normal population height as reported in several studies [7]. As normal puberty is a key point to reach normal final height, unmet needs of CKD children in the case of both delayed puberty and later onset of puberty may be the missing subjects [8, 9]. This study has focused on the age of starting EE replacement therapy as one of the key parameters for reaching near normal FH in female CKD patients with growth retardation, delayed puberty or later onset of puberty (no thelarche and no laboratory sign of pubertal onset). According to the previous investigations, puberty started in 60 and 85% of normal girls by the age 10 and 11 yrs., respectively [10]. In this study we evaluated Estrogen Therapy on final height of CKD girls with short stature who had no sign of pubertal onset until 11 yrs.

## Method

### Study design

This open label, quasi-experimental, matched controlled clinical trial was performed from 2010 till 2021, at the Ali-Asghar Children Hospital as a tertiary referral center in Tehran, Iran. Recently, this trial was also registered online in Iranian Registry of Clinical Trial with trial number IRCT20201110049334N1.

### Definitions

Stage III-IV CKD was defined as Glomerular Filtration Rate (GFR) less than 60 ml/min per 1.73 m^2^. According to CDC growth chart for stature of 2-20 years girls, persistent short stature was defined as height below 3th percentile and growth velocity of height less than 25th percentile for at least 1 year.

Pubertal onset can be defined based on hormonal levels and clinical markers (e.g., Tanner stage, growth spurt). Normal girls’ puberty usually begins between age 8–13 years (with 50 percentile of 9.74 years old). According to Tanner stages of puberty, delayed puberty was defined as lack of stage B2 thelarche until 13.5 years old or amenorrhea until 15 years old. We considered no laboratory signs of sexual maturity besides no thelarche by age 11 years as later onset of puberty because 85% of normal girls pass Tanner stage II by the age of 11 years [10].

Calcium profile consist of serum Calcium concentration (Ca), serum Phosphor concentration (Ph), Para-Thyroid Hormone (PTH) level, and Fraction excretion of Phosphor (FeP). FeP was calculated by the formula: FeP = (urine phosphate × serum creatinine) × 100/ (serum phosphate X urine creatinine).

Before enrolment and at the end of the study, bone mineral density was defined as Z score of L1-L4 for lumbar bones and Z score of neck for femur bone density in comparison with participants peers with same sex and height, by 3 Dimensioned Dual-energy X-ray Absorptiometry (3D-DXA, Stratos dR™) at Children Medical Center of Tehran. For Mid-Parental Height (MPH) of girls, father height minus 13 cm is averaged with mother height. Final height was measured when; 1. The participant’s bone age reached at least 15 years old (growth plates fusion was observed), 2. They passed 15 years of age, and 3. They had less than 0.5 cm growth during the last year of final height follow up.

### Participants

A pediatric nephrologist along with two pediatric endocrinologists evaluated all girls with stage III-IV CKD referred to nephrology clinic for determining the eligible participants. As the etiology of the CKD may relate to patients’ final heights, patients baseline disease variables were matched in 3 groups to prevent their confounding effects. All of the cases had fibrotic renal tissue at diagnostic biopsy and their pathologies of CKD were Urinary Tract Infection, Systemic Lupus Erythematous, Hypertension, Autosomal Recessive Poly Kidney Disorder, Vesico-Urethral Reflux, Hemolytic Uremic Syndrome, Diarrhea, Cystinosis, Nephrotic Syndrome, and some cases had unknown origin for their kidney failure. Totally, 70% of participants had renal and urologic congenital disorders and 30% of them had acquired glomerular disorders (Additional file 1). Metabolic acidosis, anemia, water and electrolytes imbalances, and hypothyroidism were treated with standard conventional treatments and regular monitoring was performed during the study.

Puberty was evaluated in several visits from 8 years old by measuring gonadotropic axis and clinical examinations. We followed all patients for at least 9 years and their bone age were evaluated annually by left wrist and hand radiography. Final height was defined when bone age of the participant was over 15 years. Although, bone age is not a good indicator for estimating final height in CKD children, we only used it for defining the time of stopping GH therapy and increasing external estrogen to adult dose.

Inclusion criteria were as follows: 1) Acquisition of written informed consent by patient’s legally authorized representatives and patients over 7 yrs. old, 2) Stage III-IV CKD, 3) Persistent short status before 9 years, 4) Age at the start of follow up below 7 yrs., 5) Later onset of puberty (besides prepubertal laboratory findings) or delayed puberty, 6) Girl. Exclusion criteria were as follows: 1) Lack of compliance for taking prescribed drugs or regular pediatric endocrinologist visits, 2) Renal transplantation, 3) Spontaneous onset of puberty before Ethinyl Estradiol (EE) administration and 4) Observing GH or EE specific adverse effects.

### Intervention and comparison groups

Clinical trial was performed in three arms and all included girls were on hemo- or peritoneal dialysis. All participants were evaluated at the beginning of the study, every 3 months, and at the end point of the study when they reached final height.

Participants of group 1 were treated with GH (Genotropin, Pfizer Inc., New York, NY) when growth impairment was diagnosed. GH therapy was administered subcutaneously with dose 0.05 mg/kg/day and continued until reaching height velocity lesser than 2-2.5 cm per year or age 14 yrs. These patients also received EE from 11 years old when later onset of puberty was detected. EE was started at a similar dose to patients with hypogonadism (5 μg/day, prepared as 1 ml from dissolved 50 μg EE tablet in 10 ml of water) then doubled every 3-6 months with the maximum dose 30 μg/day before growth plate closed and then finally increased gradually up to 500 μg/day.

Group 2 participants were treated with GH with the same dose as group 1, when growth impairment was diagnosed. EE was administered at the 13 years when delayed puberty was detected in these patients. EE was started at 5 μg/day (prepared as 1 ml from dissolved 50 μg EE tablet in 10 ml of water), then doubled every 3-6 months with the maximum dose 30 μg/day before growth plate closed and then finally increased gradually up to 500 μg/day. Group 3 had patients that did not accept to start GH and EE treatment until they were 15 years old and they were assigned as the control group. They just received dialysis and common relative renal failure treatments and all of them had short stature and pubertal delay.

All three groups’ participants were matched for age, the kind of dialysis, etiology of CKD, MPH, height at the beginning of the study, age of starting dialysis, and CKD stage for all groups and the age of starting GH for groups 1 and 2 to minimize the confounder effects. Management of renal failure, growth velocity evaluation, and pubertal assessment were followed carefully every 3 months in all the three groups. Bone densitometry in lumbar zone (L1-L4) and femur neck was done at the start and end of the study for all the groups. Additional bone densitometry was performed shortly after starting GH treatment and at the end of GH therapy for groups 1 and 2.

### Outcome

In this study, we aimed to identify the role of later onset of puberty, delayed puberty, and their related sexual hormone deficiencies on the FH of short stature CKD girls. So, we estimated the effect of Estrogen besides GH administration on FH, the GH therapy outcomes, bone density, and Calcium profile.

### Statistical analysis

Children were categorized based on the age of estrogen administration as explained in definitions and intervention groups section. The main outcome was final height, and secondary outcomes were calcium profile and bone densitometry.

SPSS version 16.0 software (SPSS Inc., Chicago, IL, USA) was used for statistical analysis. Continuous data were presented as mean with Standard Deviation (SD) and categorical data as absolute and their percentage. The one-way analysis of variance (One-way ANOVA) was used to determine differences between the means of more than two independent groups and when the results were significant, post hoc analysis using Tukey test was performed for pair-wise comparison of study groups. Linear regression analysis was used to assess the association between study variables and final height. Multiple linear regression analysis with backward method was used for adjust the effect of potential confounders. A *p* value of less than 0.05 was considered as significant.

## Results

### Eligible participants and demographic features

**Fig. 1:**
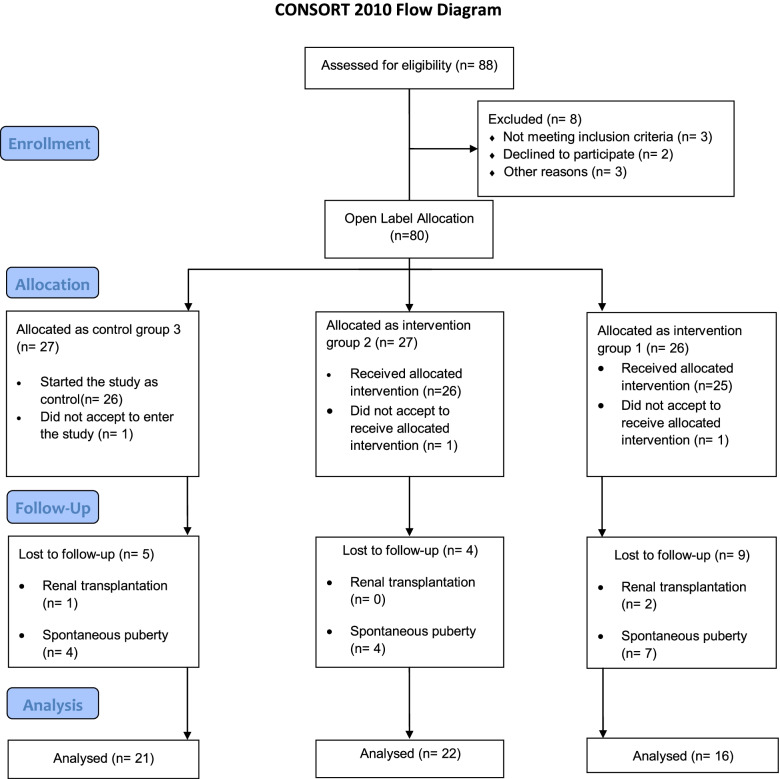
CONSORT flow diagram: Enrolment and Allocation of participants

Eighty eight girls were diagnosed as stage III-IV CKD in the nephrology clinic, and out of these, 80 were initially included in this trial (Fig. 1). We allocated them to three groups after matching their basic characteristics. In total, 21 patients were excluded due to followed reasons; 1) 3 patients did not accept to give informed consent, 2) 15 patients had spontaneous puberty, 3) and 3 patients received renal transplantation. Finally, 16, 22, and 21 patients were allocated to groups 1, 2, and 3 respectively (Fig. 1). All included girls were on hemo- or peritoneal dialysis from 3 to 5.5 years age and groups 1 and 2 were treated with GH from 7.5– 9.2 years of age when their growth impairment was diagnosed.

The characteristics of participants are summarized in Table 1 with respect to study groups. Mean MPHs in groups 1, 2, and 3 were 159.5 (± 2), 161.3 (± 3.2), and 160.3 (± 3) respectively. Group 1 received GH for 37.8 (±6) months before EE administration at the age of 11 yrs. and group 2 received GH for 52.9 (±8) months before EE administration at the age of 13 yrs. Mean bone density Z score and calcium profile levels are illustrated in Table 1.

**Table 1:** Baseline characteristics of each group before intervention and over 15 years old and bone age 15 years (Mean ± SD)

Variables	Group 1 (*n* = 16)	Group 2 (*n* = 22)	Group 3 (*n* = 21)	*P* value
FH (cm)	154.5 ± 1.4^a^	151.6 ± 2.3^b^	146.1 ± 2.3^c^	< 0.001
FH SD score^a^	−1.14 ± 0.22^a^	−1.59 ± 0.36^b^	−2.44 ± 0.36^c^	< 0.001
MPH (cm)	159.5 ± 2	161.3 ± 3.2	160.3 ± 3	0.15
GH therapy before EE (month)	37.8 ± 6	52.9 ± 8	–	–
Lumbar (L1-L4) Density Z Score				
Primary	−3 ± 0.5	–	–	
Final	−2.3 ± 0.3^a^	−2.8 ± 0.5^b^	−3.1 ± 0.6^b^	< 0.001
Femur Head Density Z Score				
Primary	−3.5 ± 0.7	−3.4 ± 0.7	–	–
Final	− 2.6 ± 0.4^a^	−2.9 ± 0.4^a^	−3.5 ± 0.5^b^	< 0.001
Ca (mg/dl)				
Primary	8.7 ± 0.2	8.7 ± 0.2	–	–
Final	8.8 ± 0.1^a^	8.7 ± 0.2^a^	8.4 ± 0.2^b^	< 0.001
Ph (mg/dl)				
Primary	6.9 ± 0.4	6.7 ± 0.5	–	–
Final	6.2 ± 0.3^a^	5.9 ± 0.3^b^	7.4 ± 0.4^c^	< 0.001
PTH (pg/ml)				
Primary	166 ± 0.2	176 ± 0.1	–	–
Final	143 ± 27^a^	155 ± 27^a^	279 ± 49^b^	< 0.001
FeP (%)				
Primary	4.6 ± 0.1	4.9 ± 0.1	–	–
Final	8.3 ± 1.9	8.6 ± 2.5	8.4 ± 0.2	0.862

One-Way ANOVA was used for Value analysis

Value presented as mean ± SD

In each row dissimilar values (a, b, c) are significantly different

^a^Final Height SD score according to the CDC growth chart for stature of 2-20 years old girls

### Final height and bone density

In one-way analysis of variance (one-way ANOVA), mean MPH had no significant difference between groups 1, 2, and 3 (*p* value: 0.15). GH therapy significantly enhanced mean final height in groups 1 (154.5 ± 1.4 cm and − 1.14 ± 0.22 SD score) and 2 (151.6 ± 2.3 cm and − 1.59 ± 0.36 SD score) in comparison with group 3 (146.1 ± 2.3 cm and − 2.44 ± 0.36 SD score) (*p* value: < 0.001).

Also, we found girls were significantly taller in group 1 than group 2 (*p* value: < 0.001), so, EE administration in 11 yrs. old CKD girls with short stature and later onset of puberty enhanced final height in comparison with the EE administration in 13 yrs. old (Table 1).

Moreover, in one-way analysis of variance (ANOVA), mean femoral bone density was significantly enhanced after GH and EE treatment in both groups 1 (− 2.6 ± 0.4) and 2 (− 2.9 ± 0.4) in comparison with group 3 (− 3.5 ± 0.5) (*p* value: < 0.001). Mean lumbar bone density of group 1 (− 2.3 ± 0.3) was significantly more than group 2 (− 2.8 ± 0.5) and group 3 (− 3.1 ± 0.6) (*p* value: < 0.001). So, EE administration in 11 yrs. old CKD girls with short stature and later onset of puberty improved lumbar bone density in comparison with EE administration in 13 yrs. old or later (Table 1).

### Calcium profile

In one-way ANOVA, calcium profile had no significant clinical and statistical difference between groups 1 and 2 (Table 1). Mean serum Ca increased significantly in group 1 (8.8 ± 0.1) and group 2 (8.7 ± 0.2) in comparison with group 3 (8.4 ± 0.2) (*p* value: < 0.001). Also, mean PTH had a significant reduction in group 1 (143 ± 27) and group 2 (155 ± 27) in comparison with group 3 (279 ± 49, *p* value: < 0.001) (Table 1).

Additionally, when we analyzed calcium profile in group 1, mean serum Ca was not significantly different before and after intervention. But GH and EE therapy significantly reduced mean serum Ph (*p* value: < 0.001) and mean PTH (*p* value: 0.005) and also significantly enhanced mean FeP (*p* value: < 0.001).

In calcium profile of group 2 before and after intervention, mean serum Ca was not significantly different. However, GH and EE therapy significantly reduced mean serum Ph (*p* value: < 0.001) and mean PTH (*p* value: < 0.001) and also significantly enhanced mean FeP (*p* value: < 0.001) in group 2.

Therefore, GH and EE administration at appropriate time significantly improved Ca profile (reduced mean serum Ph and mean PTH, and also enhanced mean FeP) in CKD girls with short stature and delayed puberty.

### Linear regression analysis for final height

In univariable linear regression analysis (Model 1), we found significant positive association between final height and age of starting GH (β = 0.19, *p* = 0.012), height of starting GH (β = 0.18, *p*: 0.06), paternal height (β = 0.29, *p*: 0.034), and duration of GH therapy after starting EE (β = 0.15, *p* < 0.001). Also, there was significant negative association between final height and all the 3 groups (β = − 4.29, *p* < 0.001), duration of GH therapy before starting EE (β = − 0.12, *p* < 0.001), age of starting EE (β = − 0.15, *p* < 0.001), and height of starting EE (β = − 0.14, *p*: 0.005) (Table 2).

**Table 2:** Multiple linear regression analysis assessed association between study variables and the final height in different groups of CKD girls with short stature and delayed puberty

Variables	Univariable linear regression Beta (95%CI)	*P* value	Adjusted model^a^ Beta (95%CI)	*P* value	Backward linear regression Beta (95%CI)	*P* value
Groups	−4.29 (−5.02/−3.57)	< 0.001	−0.79 (− 4.41/2.82)	0.657	–	–
Age of starting GH	0.19 (0.04/0.33)	0.012	−0.042(− 0.18/0.09)	0.549	–	–
Height of starting GH	0.18 (−0.008/0.38)	0.06	0.27 (0.05/0.48)	0.016	0.22 (0.09/0.35)	0.001
Paternal Height	0.29 (0.02/0.55)	0.034	0.15 (0.03/0.27)	0.013	0.14 (0.04/0.25)	0.009
MPH	0.14 (−0.21/0.51)	0.419	–	–	–	–
Maternal Height	−0.07 (− 0.38/0.23)	0.648	–	–	–	–
Duration of GH Therapy	–	–	–	–	–	–
Duration of GH Therapy before starting EE	−0.12 (− 0.19/− 0.06)	< 0.001	0.07 (0.007/0.14)	0.033	0.07(0.007/0.14)	0.031
Age of starting EE	−0.15 (− 0.21/− 0.10)	< 0.001	−0.23 (− 0.41/− 0.05)	0.011	−0.26 (− 0.37/− 0.15)	< 0.001
Height of starting EE	−0.14 (− 0.24/− 0.04)	0.005	0.20 (− 0.003/0.4)	0.053	0.19 (0.01/0.37)	0.035
Duration GH therapy after starting EE	0.15 (0.09/0.22)	< 0.001	0.12 (0.03/0.22)	0.011	0.11 (0.02/0.20)	0.011
Age of Discontinuing GH	0.07 (−0.09/0.23)	0.395	–	–	–	–

^a^Adjusted linear regression: All variables with *p* value less than 0.2 were included in the model

## Discussion

In this clinical trial, short stature CKD girls with later onset of puberty and delayed puberty significantly benefited from EE administration at 11 yrs. age in comparison with those at 13 yrs. Both final height and lumbar bone density improved significantly when EE was started at 11 yrs. age in comparison with the 13 yrs. old.

Growth impairment is a critical challenge in pediatric CKD [11] and even after renal transplantation children struggle with short stature [12, 13]. In healthy children, 80% of growth has already been achieved before puberty. However, pubertal growth spurt is an important parameter to reach an acceptable final height. Gonadotropic hormones axis stimulates growth during puberty via increased proliferation of growth plate chondrocytes and modulation of GH secretion from the pituitary gland and this axis causes the pubertal growth spurt [7, 14]. The main factor for pubertal growth spurt of girls is estrogen hormone [14].

Approximately, 50% of children requiring renal replacement therapy before their 13th birthday show delayed puberty and have a final height below the normal range [13]. In these patients, pubertal growth spurt starts with a lower mean velocity and its duration is approximately 1.5 years (1 year shorter than normal girls) [12]. Estrogen itself and its effect on increasing insulin like growth factor 1 (IGF1) and GH release, are important factors for bone maturation and so delayed puberty is a major risk factor for growth retardation, osteoporosis, and reduced final height [15].

Puberty is a time consuming process and estrogen as the main sexual hormone in girls, increases slowly during 2 years to reach its maximum level [16]. Finally, high concentration of estrogen makes growth plate completely mature and closed.

According to the international Turner Syndrome guidelines, it is recommended to initiate estrogen therapy when patients are between 11 and 12 years of age, and then gradually increasing the external estrogen to adult dosage over 2 to 3 years [17]. In this manner of estrogen administration, it mimics the normal estrogen enhancement of puberty and may resume growth spurt in these patients. Also, combination of GH therapy with very low-dose estrogen in girls with Turner syndrome has shown enhancement in growth response [18].

Endocrinologists recommended administering low dose estrogen from 11 years age in the cases that are identified as pubertal failure, such as Turner syndrome to improve their life style and bone density [19, 20]. CKD is identified as an etiology for delayed puberty because it decreases the mass of bioactive and immune-active LH secreting and disturbing gonadotropic hormones axis [14, 16, 19].

Pfizer International Growth Database board designed a cohort on 240 CKD children and they illustrated the importance of puberty onset in growth of the CKD girls. It was found that GH therapy enhanced CKD girls’ mean height SD score until final height by 1.6 [21]. They also found that the time of puberty onset had a significant role in this enhancement. Therefore, if the GH has been started in pre-pubertal CKD girls with normal onset of puberty, the mean final height was − 2 SD, while if GH has been administered in girls with severely delayed puberty, the mean final height was − 3.6 SD. In this study we investigated the effect of EE replacement therapy for later onset of puberty in CKD girls with short stature. Participants of both groups 1 and 2 received GH therapy from 7.5-9 years old but EE therapy was started at 11 and 13 years old respectively. The results illustrated that low dose EE therapy beginning at 11 years old could enhanced mean final height by near 3 cm and height SD score until final height by 0.45.

So we propose that CKD girls who suffered from both short stature and delayed puberty could benefit from GH and EE therapy at the most proper time to improve their final height, bone density and Ca profile (reduced mean serum Ph and mean PTH, and also enhanced mean FeP). As mentioned in the results of our study, starting low dose estrogen replacement therapy at 11 years age could improve mean final height by 0.5 SD in comparison with the 13 years old and 1.3 SD in comparison with the girls with no GH and EE therapy.

## Conclusion

Clinicians may neglect later onset of puberty in girls and so miss the effect of low dose of estrogen on pubertal growth spurt. Estrogen administration for delayed puberty after 13 - 13.5 yrs. old in girls, leads to missing the critical effect of estrogen minimal dose during the process of puberty.

As GH therapy is a long and expensive treatment, we recommended starting low dose estrogen replacement therapy (by using the same protocol as girls with pubertal failure) in short stature girls with CKD at 11 yrs. that have no clinical (thelarche) and laboratory sign of onset of sexual maturity until that age to enhance the cost effectiveness of GH therapy.

## Strengths and limitations

The first limitation is that our clinical trial had no blinding or randomization. In the case of CKD, we could not convince patients and their legally authorized representatives to accept unknown intervention. We matched participants in each group to minimize the confounder effects. Also, we chose three groups to find the exact effect of EE besides GH on final height.

The second limitation is that for the participants of groups 1 and 2, GH therapy could not be started at the same age. In addition, although Ali-Asghar children hospital is a tertiary referral center in Tehran, the number of CKD short statured pediatric cases with delayed puberty were limited and so, we could not perform the study if we limited the GH therapy at a definite age. Therefore, we propose multi-centric study with larger sample size to evaluate the effect of age of starting EE on CKD short statured girls with delayed puberty that have started GH at a definite age.

## Supplementary Information

**Additional file 1.** CKD basal characteristics of participants.

## Data Availability

The data that support the findings of this study are available on request from the corresponding author, Dr. Sedigheh Madani.

## Abbreviations

FH: Final Height
CKD: Chronic Kidney Disease
GH: Growth Hormone
EE: Ethinyl Esradiol
MPH: Mead Parental Height
PTH: Para Thyroid Hormone
ESRD: End Stage Renal Disorders
GFR: Glomerular Filtration Rate
FeP: Fraction excretion of Phosphor
Ca: Calcium
Ph: Phosphor
SD: Standard Deviation
ANOVA: Analysis of Variance
