# Supplementary material for: Estrogen replacement therapy: effects of starting age on final height of girls with chronic kidney disease and short stature
Source: BMC Pediatr. 2022 Jun 21;22:355. doi: 10.1186/s12887-022-03406-y (PMC9210764; doi:10.1186/s12887-022-03406-y)
Supplement: Supplementary file 1 — Additional file 1. CKD basal characteristics of participants. [file 12887_2022_3406_MOESM1_ESM.docx]

Additional file 1

CKD basal characteristics of participants.

| group | No. | Pathology | Age of CKD (month) | Age of starting Dialysis (month) | Kind of Dialyses |
| --- | --- | --- | --- | --- | --- |
| 1 |  | AR-PKD | 7 | 21 | H&P |
|  |  | SLE | 36 | 54 | H |
|  |  | HTN | 54 | 72 | H |
|  |  | UTI | 72 | 84 | H |
|  |  | VUR | 48 | 66 | H |
|  |  | UTI | 66 | 102 | H |
|  |  | CYSTINOSIS | 11 | 39 | P |
|  |  | UTI | 16 | 36 | H |
|  |  | CYSTINOSIS | 9 | 33 | H |
|  |  | AR-PKD | 10 | 30 | H |
|  |  | UTI | 54 | 72 | P |
|  |  | VUR | 27 | 45 | P |
|  |  | UTI | 39 | 57 | H |
|  |  | VUR | 21 | 45 | H&P |
|  |  | VUR | 39 | 57 | H |
|  |  | VUR | 33 | 60 | H |
| 2 |  | DIARRHEA | 30 | 36 | P |
|  |  | CYSTINOSIS | 9 | 27 | H |
|  |  | DIARRHEA | 36 | 48 | H |
|  |  | UTI | 48 | 57 | H |
|  |  | HUS | 42 | 63 | H&P |
|  |  | VUR | 48 | 63 | P |
|  |  | SLE | 18 | 33 | H&P |
|  |  | SLE | 30 | 42 | H |
|  |  | HTN | ≺36 | 36 | H |
|  |  | AR-PKD | 8 | 39 | H |
|  |  | VUR | 15 | 42 | H&P |
|  |  | DIARRHEA | 39 | 51 | H |
|  |  | VUR | 15 | 42 | P |
|  |  | AR-PKD | 4 | 60 | H |
|  |  | CYSTINOSIS | 6 | 45 | P |
|  |  | UTI | 18 | 37 | H |
|  |  | VUR | 31 | 48 | H |
|  |  | Unknown | 14 | 31 | P |
|  |  | AR-PKD | 8 | 27 | H |
|  |  | UTI | 19 | 42 | H |
|  |  | VUR | 38 | 54 | H |
|  |  | UTI | 27 | 59 | H |
|  |  | AR-PKD | 5 | 14 | H |
|  |  | UTI | 20 | 45 | P |
|  |  | VUR | 35 | 51 | H |
|  |  | VUR | 41 | 64 | H |
|  |  | CYSTINOSIS | 11 | 35 | H |
|  |  | UTI | 52 | 69 | H |
|  |  | VUR | 48 | 65 | H |
|  |  | DIARRHEA | 34 | 54 | H |
|  |  | UTI | 21 | 47 | H |
|  |  | SLE | 31 | 63 | H |
|  |  | AR-PKD | 6 | 14 | P |
|  |  | VUR | 18 | 36 | H&P |
|  |  | VUR | 34 | 60 | H |
|  |  | Unknown | ? | 45 | H |
|  |  | VUR | 25 | 49 | P |
|  |  | Unknown | ? | 38 | H&P |
|  |  | UTI | 17 | 35 | H |
|  |  | VUR | 34 | 62 | P |
|  |  | SLE | 22 | 43 | H |
|  |  | HUS | 23 | 48 | H |
|  |  | DIARRHEA | 18 | 53 | H |

VUR: Vesicourethral Reflux

UTI: Urinary Tract Infection

SLE: Systemic Lupus Erythematous

AR-PKD: Autosomal Recessive poly cystic Kidney Disease
